# Influenza A (H10N7) Virus Causes Respiratory Tract Disease in Harbor Seals and Ferrets

**DOI:** 10.1371/journal.pone.0159625

**Published:** 2016-07-22

**Authors:** Judith M. A. van den Brand, Peter Wohlsein, Sander Herfst, Rogier Bodewes, Vanessa M. Pfankuche, Marco W. G. van de Bildt, Frauke Seehusen, Christina Puff, Mathilde Richard, Ursula Siebert, Kristina Lehnert, Theo Bestebroer, Pascal Lexmond, Ron A. M. Fouchier, Ellen Prenger-Berninghoff, Werner Herbst, Marion Koopmans, Albert D. M. E. Osterhaus, Thijs Kuiken, Wolfgang Baumgärtner

**Affiliations:** 1 Department of Viroscience, Erasmus Medical Center, Wytemaweg 80, 3015 CN, Rotterdam, the Netherlands; 2 Department of Pathology, University of Veterinary Medicine Hannover, Bünteweg 17, 30559, Hannover, Germany; 3 Institute for Terrestrial and Aquatic Wildlife Research (ITAW), University of Veterinary Medicine Hannover, Werftstraβe 6, D-25761, Büsum, Germany; 4 Institute for Hygiene and Infectious Diseases of Animals, Justus-Liebig-University, Frankfurter Straβe 85-89, 35392, Giessen, Germany; St. Jude Children's Research Hospital, UNITED STATES

## Abstract

Avian influenza viruses sporadically cross the species barrier to mammals, including humans, in which they may cause epidemic disease. Recently such an epidemic occurred due to the emergence of avian influenza virus of the subtype H10N7 (Seal/H10N7) in harbor seals (*Phoca vitulina*). This epidemic caused high mortality in seals along the north-west coast of Europe and represented a potential risk for human health. To characterize the spectrum of lesions and to identify the target cells and viral distribution, findings in 16 harbor seals spontaneously infected with Seal/H10N7 are described. The seals had respiratory tract inflammation extending from the nasal cavity to bronchi associated with intralesional virus antigen in respiratory epithelial cells. Virus infection was restricted to the respiratory tract. The fatal outcome of the viral infection in seals was most likely caused by secondary bacterial infections. To investigate the pathogenic potential of H10N7 infection for humans, we inoculated the seal virus intratracheally into six ferrets and performed pathological and virological analyses at 3 and 7 days post inoculation. These experimentally inoculated ferrets displayed mild clinical signs, virus excretion from the pharynx and respiratory tract inflammation extending from bronchi to alveoli that was associated with virus antigen expression exclusively in the respiratory epithelium. Virus was isolated only from the respiratory tract. In conclusion, Seal/H10N7 infection in naturally infected harbor seals and experimentally infected ferrets shows that respiratory epithelial cells are the permissive cells for viral replication. Fatal outcome in seals was caused by secondary bacterial pneumonia similar to that in fatal human cases during influenza pandemics. Productive infection of ferrets indicates that seal/H10N7 may possess a zoonotic potential. This outbreak of LPAI from wild birds to seals demonstrates the risk of such occasions for mammals and thus humans.

## Introduction

In 2014 and the beginning of 2015, there was an epidemic of a novel H10N7 influenza A virus (Seal/H10N7) among harbor seals (*Phoca vitulina*) resulting in increased mortalities along the coasts of Sweden, Denmark, Germany and The Netherlands [1–5]. Most probably this epidemic was caused by a spill-over of a low pathogenic avian influenza virus (LPAI) originating from wild aquatic birds, since this Seal/H10N7 is genetically closely related to various influenza viruses detected in wild birds [3, 5].

Spill-over events of influenza A viruses from wild water-birds to seals have been reported on different occasions as reviewed by White [6]. In 2011 there was an outbreak of fatal H3N8 influenza virus infection in harbor seals on the coast of New England, United States. This H3N8 virus is most likely of avian origin and the results of some studies suggest adaption to humans by increased binding affinity for the human-type α2,6-sialic acid receptors [7, 8], while others do not [9]. In seals this virus caused interstitial pneumonia, hemorrhagic alveolitis and necrotizing bronchitis. Virus antigen and RNA was found in bronchiolar epithelium by immunohistochemistry (IHC) and *in situ*-hybridization (ISH) [7, 10]. Ferrets infected with this H3N8 virus demonstrated virus titers of above 10^6^ TCID^50^/g in the nasal turbinates and the lungs [11]. The few pathological investigations on seals that died during this Seal/H10N7 outbreak report similar lesions, consisting of necro-suppurative bronchopneumonia with a bacterial co-infection [1, 2]. The whole spectrum of virus-associated histological changes, the distribution of viral antigen and the target cell tropism of Seal/H10N7 in harbor seals are unknown. This study provides a more comprehensive description of histological, IHC, virological, bacteriological and parasitological findings in 16 naturally infected seals that were described previously by Bodewes and co-workers [3, 5]. Since the zoonotic potential of Seal/H10N7 is unknown, especially people handling ill seals, performing necropsies, or disposing carcasses may be at risk. A good and well accepted animal model for severe respiratory disease caused by influenza A viruses in humans is the ferret after intratracheal inoculation. Therefore, to predict the cell tropism and lesions solely caused by Seal/H10N7 in seals (and humans) without bacterial co-infection, six ferrets were intratracheally inoculated with Seal/H10N7, euthanized on 3 or 7 days post infection, necropsied and sampled for pathology, IHC and virology.

## Materials & Methods

### Naturally infected harbor seals with Seal/H10N7

In October 2014, 2 adult (1 male, 1 female) and 14 juvenile (11 male, 3 female) harbor seals (case nos. 1–16; Table 1) were selected for this study based on a positive PCR for influenza A virus a tracheal/throat swab or in a lung tissue sample from a larger collection of dead or moribund seals found along the German North Frisian coast and the island of Helgoland. Except one juvenile individual that had been euthanized, all other seals had died spontaneously. After retrieval from the coast the carcasses were kept at +4°C or were deep frozen (-80°C) until necropsy (Table 1). A short report about the findings and molecular identification of the virus has been published previously [3, 5]. A complete necropsy was performed on each seal. Numerous organ and tissue samples were collected including skin, skeletal muscle, tongue, mandibular, retropharyngeal, pulmonary and mesenteric lymph nodes, tonsils, bone marrow, thymus, nasal mucosa, trachea, lung, heart, aorta, spleen, liver, pancreas, kidneys, urinary bladder, esophagus, stomach, small and large intestine, gonads, uterus, adrenal glands, thyroid glands, brain and spinal cord. The samples were fixed in 10% neutral-buffered formalin and processed routinely into paraffin wax.

**Table 1:** Postmortem interval, histological diagnosis in the lungs, immunohistochemical findings in respiratory tracts, microbiological findings and severity of parasitic infestation in the lungs of seals naturally infected with Seal/H10N7. PMI = approximate post mortem interval (days); *carcass had been deep frozen; (f) = carcass stored a +4°C until necropsy; n. k. = date of carcass retrieval not known. Histological diagnosis: foc = focal; of = oligofocal; mf = multifocal; eos = eosinophilic; + = mild; ++ = moderate; +++ = severe. Immunohistological findings: +++ = many cells; ++ = moderate number of cells; + = few cells; +/- = occasional cells; not examined = no tissue preserved. S. = Streptococcus; B. = Bordetella.

Animal	PMI (days)	Diagnosis (lung)	Influenza virus antigen immunolabelling				*Mycoplasma* PCR	Bacteriological culture	Lungworm infestation
			Nasal mucosa	Tracheal mucosa	Lung				
					Bronchial mucosa	Bronchial glands			
1	n. k.*	mf ++ necrosuppurative pneumonia/adenitis	positive (+/-)	negative	negative	positive (+/-)	negative	*S*. *equi* ssp. *zooepidemicus*	-
2	3*	mf +++ suppurative and granulomatous pneumonia	negative	positive (+/-)	positive (++)	positive (+)	positive	*S*. *phocae*	+
3	3*	mf +++ suppurative to granulomatous/eos pneumonia	positive (+/-)	negative	negative	negative	positive	*B*. *bronchiseptica*	++
4	3 (f)	mf ++/+++ necro-suppurative and granulomatous pneumonia	positive (+/-)	negative	positive (++)	positive (++)	positive	*B*. *bronchiseptica*	++/+++
5	n. k. (f)	mf +/++ necrosuppurative, mf ++ granulomatous/eos pneumonia	negative	positive (+)	positive (++)	positive (+)	negative	no significant bacterial growth	+++
6	n. k.*	mf ++/+++ necrosuppurative and granulomatous pneumonia/adenitis	positive (+/-)	positive (+/-)	positive (+)	positive (++)	negative	*S*. *phocae*, *Escherichia coli*	+++
7	3 (f)	mf ++/+++ granulomatous pneumonia	negative	negative	negative	negative	positive	*B*. *bronchiseptica*	+++
8	13*	mf +++ suppurative pneumonia	not examined	negative	negative	negative	negative	*S*. *equi ssp*. *zooepidemicus*	-
9	12*	mf ++ granulomatous/eos pneumonia	positive (+)	positive (+/-)	positive (+)	positive (+)	negative	*S*. *phocae*, *Pseudomonas* spp., *Moraxella* spp.	++
10	n. k.*	mf ++ granulomatous/eos pneumonia	negative	negative	positive (+/-)	negative	negative	*S*. *phocae*	+/++
11	6*	of ++/+++ granulomatous/necrotizing/eos pneumonia	not examined	positive (+/-)	positive (++)	positive (+/-)	negative	*Brucella* spp.	+++
12	7*	foc +++ necrosuppurative bronchopneumonia/adenitis	not examined	positive (+)	positive (+)	positive (++)	negative	*S*. *equi* ssp. *zooepidemicus*, *S*. *phocae*	+++
13	1 (f)	mf ++ granulomatous/eos; mf +/++ bronchointerstial pneumonia	positive (+++)	positive (+)	positive (+)	positive (++)	negative	*S*. *phocae*, alpha-hemolysing *Streptococci*	+
14	1 (f)	foc + granulomatous to eosinophilic and mf + bronchointerst pneumonia	negative	negative	negative	negative	negative	*Arcanobacerium phocae*, *Neisseria* spp., *Corynebacterium* spp.	+/++
15	5*	mf ++/+++ granulomatous/eos and mf + bronchointerstitial pneumonia	not examined	negative	negative	negative	negative	no significant bacterial growth	+/++
16	3 (f)	mf +/++ granulomatous, foc +++ fibrino-suppurative pneumonia/adenitis	negative	positive (+/-)	positive (+)	positive (++)	negative	*S*. *phocae*, *E*. *coli*	+

For histopathology, 3–5 μm thick paraffin sections were stained with hematoxylin and eosin (HE). IHC on paraffin-embedded tissue sections of all organs was performed using the avidin-biotin-peroxidase complex (ABC) method. For the detection of morbillivirus nucleoprotein, a polyclonal antibody (Lot 25, rabbit #162) was applied as described previously [12]. A monoclonal antibody (HB65) was used for immunolabelling of influenza A nucleoprotein [13]. For negative control purposes, the primary antibody was replaced by ascitic fluid from non-immunized Balb/cJ mice.

Transmission electron microscopy was performed using pop-off technique on paraffin sections from lung tissue taken exemplary from seal no. 16 as described previously[14]. Real-time RT-qPCR targeting the influenza A virus matrix gene was performed on lung and tracheal swab samples collected from the seals as described previously [3, 5].

Samples for microbiological investigations were taken under abacterial conditions from lung, pulmonary lymph node, liver, kidney, spleen, intestine and mesenteric lymph node. The samples were frozen at –25°C until further processing. For cultivation, samples were decontaminated superficially by heat after thawing and a new cut surface was streaked on blood agar containing 5% defibrinated sheep blood (E. Merck, Darmstadt, Germany) and water-blue metachrome-yellow lactose agar (acc. to Gassner, E. Merck). Plates were incubated at 37°C and analysed after 24 and 48 hours. Grown colonies were subcultured and pure cultures were identified using standard morphological and biochemical methods as well as MALDI-TOF mass spectrometry with the Microflex LT/SH instrument as instructed by the manufacturer Bruker Daltonics [15]. Additionally, thiosulfate-citrate-bile-sucrose agar (TCBS) (Vibrio selective) agar (E. Merck), Brucella agar base with Brucella selective supplement (Oxoid, Wesel, Germany), KIMMIG agar (E. Merck) for selective cultivation of fungi as well as a selective medium for the isolation of *Erysipelothrix rhusiopathiae* (mod. by Böhm 1971 [16]) were incubated according to the respective methods necessary for cultivation of the different organisms (for example Brucella selective agar was incubated in a CO_2_-incubator with 10% C0_2_ for at least five days; for isolation of fungi KIMMIG agar incubation occurred at 28–30°C for three to fourteen days) [15, 17–19]. For the detection of *Mycoplasma* spp. a genus-specific PCR targeting the 16S rDNA was designed according to Rawadi and Dussurget [20].

Parasites were collected during necropsies, preserved in 70% ethanol and identified microscopically after preparation in lactophenol [21].

### Virus isolation and titration

A/harbor seal/Germany/PV20762_TS/2014 virus was isolated from a tracheal swab of a naturally infected seal (no. 9) [3] during the outbreak in Germany by passaging two times over Madin-Darby canine kidney (MDCK) cells. Virus end-point titrations were performed as described previously [22]. Briefly, MDCK cells were inoculated with tenfold serial dilution of virus stocks, nasal swabs, pharyngeal swabs and homogenized tissue samples. Cells were washed with phosphate-buffered saline solution (PBS) one hour after inoculation and cultured in infection medium, consisting of Eagle’s minimum essential medium (EMEM) supplemented with 100 U/ml penicillin, 100 μg/ml streptomycin, 2 mM L-glutamine, 1.5 mg/ml NaHC0_3_, 10 mM 4-(2-hydroxyethyl)-1-piperazineethanesulfonic acid (HEPES), 1X non-essential amino acids (NEAA), and 20 μg/ml trypsin (Lonza). Three days after inoculation, supernatants of cell cultures were tested for agglutinating activity using turkey erythrocytes as an indicator of virus replication in the cells. Infectious virus titers were calculated in quadruplicates of each of the nasal swabs, pharyngeal swabs, and homogenized tissue samples and for ten-fold replicates of the virus stocks by the method of Reed and Munch [23].

### Experimentally infected ferrets with Seal/H10N7

Six 12-month-old purpose-bred female ferrets (*Mustela putorius furo*), seronegative for currently circulating influenza A (pH1N1 and H3N2) and B viruses and Aleutian disease virus, weighing 600–900 g., were obtained from a commercial breeder (Euroferret). Animals were housed and they received water and food ad libitum. The experiments were conducted in strict compliance with European guidelines (EU directive on animal testing 86/609/EEC) and Dutch legislation (Experiments on Animals Act, 1997). The protocol was approved by an independent animal experimental ethical review committee DCC in Driebergen, The Netherlands. Animal welfare was monitored on a daily basis. Virus inoculation of ferrets was performed under anesthesia with a mixture of ketamine/medetomidine (10 and 0.05 mg/kg resp.) antagonized by atipamezole (0.25 mg/kg). All animal handling (swabbing and weighing) was performed under light anesthesia using ketamine to minimize animal suffering. All experiments with ferrets were performed under animal biosafety level 3 conditions in class 3 isolator cages.

The ferrets were inoculated intratracheally with 10^6^ median tissue culture infectious dose (TCID_50_) of Seal/H10N7 in a 3-ml volume. Clinical scores were assessed daily and the activity status was scored as follows: 0, alert and playful; 1, alert and playful only when stimulated; 2, alert but not playful when stimulated; 3, neither alert nor playful when stimulated. For diarrhea, sneezing, nasal discharge, inappetence and dyspnea we scored: 0, not present; 1, present. Dyspnea was characterized by open-mouth breathing with exaggerated abdominal movement. Body weight was monitored daily. Humane endpoints in case of severe disease prior to the experimental endpoint were set to 20% of weight loss over the course of the experiment, 15% weight loss in a day or activity score 3. Nasal and pharyngeal swabs were collected every day and were stored at -80°C in transport medium (Hank’s balanced salt solution containing 10% of glycerol, 200U/ml P, 200 mg/ml S, 100U/ml polymyxin B sulphate (Sigma Aldrich) and 250 mg/ml gentamycin [ICN, Netherlands]) until end-point titration in MDCK cells. At 3 and 7 dpi, three animals were euthanized by exsanguination. Necropsies were performed according to a standard protocol [24] and the following samples were taken for virological, histopathological and immunohistochemical analyses: nasal turbinates, trachea, bronchus, lung, tracheo-bronchial lymph nodes, tonsils, heart, liver, spleen, kidneys, adrenal glands, pancreas, jejunum, olfactory bulb, cerebellum and cerebrum. Tissues for virological examination were homogenized in transport medium using the FastPrep system (MP Biomedicals) with 2 one-quarter-inch ceramic sphere balls, centrifuged at 1500 *g* for 10 min, aliquoted and stored at -80°C until end-point titration in MDCK cells. Tissues for histological examination were fixed in 10% neutral-buffered formalin, embedded in paraffin wax, sectioned at 4 μm, and stained with HE for light microscopical examination. For detection of influenza A virus antigen by immunohistochemistry, sequential slides of all tissues were stained with a primary antibody against the influenza A nucleoprotein as described previously [25]. In each staining procedure, an isotype control was included as a negative control and a lung section from a cat infected experimentally with high pathogenic influenza virus (HPAI) H5N1 was used as positive control [26].

## Results

### Naturally infected harbor seals with Seal/H10N7

The seals were in variable nutritional conditions, ranging from very poor to good. Gross findings in spontaneously dead animals comprised poorly retracted lungs with severe congestion, alveolar and interstitial emphysema, alveolar edema, occasional diffuse consolidation, and multifocal firm nodular areas of grey-yellow discoloration with varying numbers of metazoan parasites. Other organs and tissues did not display significant changes.

**Fig 1:**
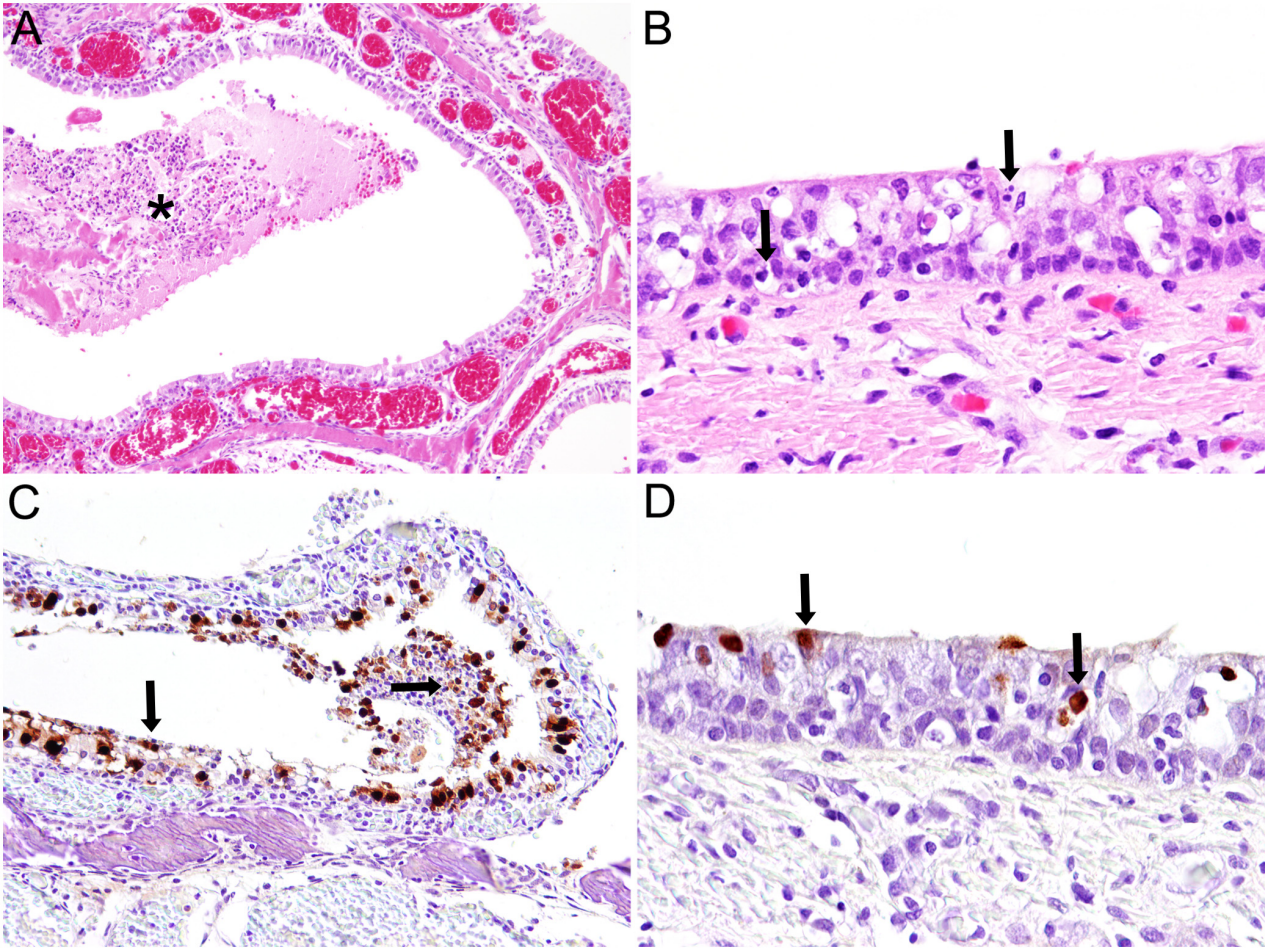
Histopathology and immunohistochemistry of nasal respiratory mucosa and tracheal mucosa of a harbor seal (No. 13) naturally infected with Seal/H10N7. Histopathologically, there was marked hyperemia (dilated blood vessels) in the nasal respiratory mucosa and accumulation of exudate (asterisk) in the nasal cavity (A; magnification 100x). In the tracheal epithelium there were numerous necrotic epithelial cells (arrows) and mild inflammatory infiltration (arrow head) (B; magnification 400x). Immunohistochemistry revealed exceptional high amounts of influenza A virus antigen (arrows) in nasal epithelial cells (C; magnification 100x) and in few tracheal epithelial cells (arrows) (D; magnification 400x).

**Fig 2:**
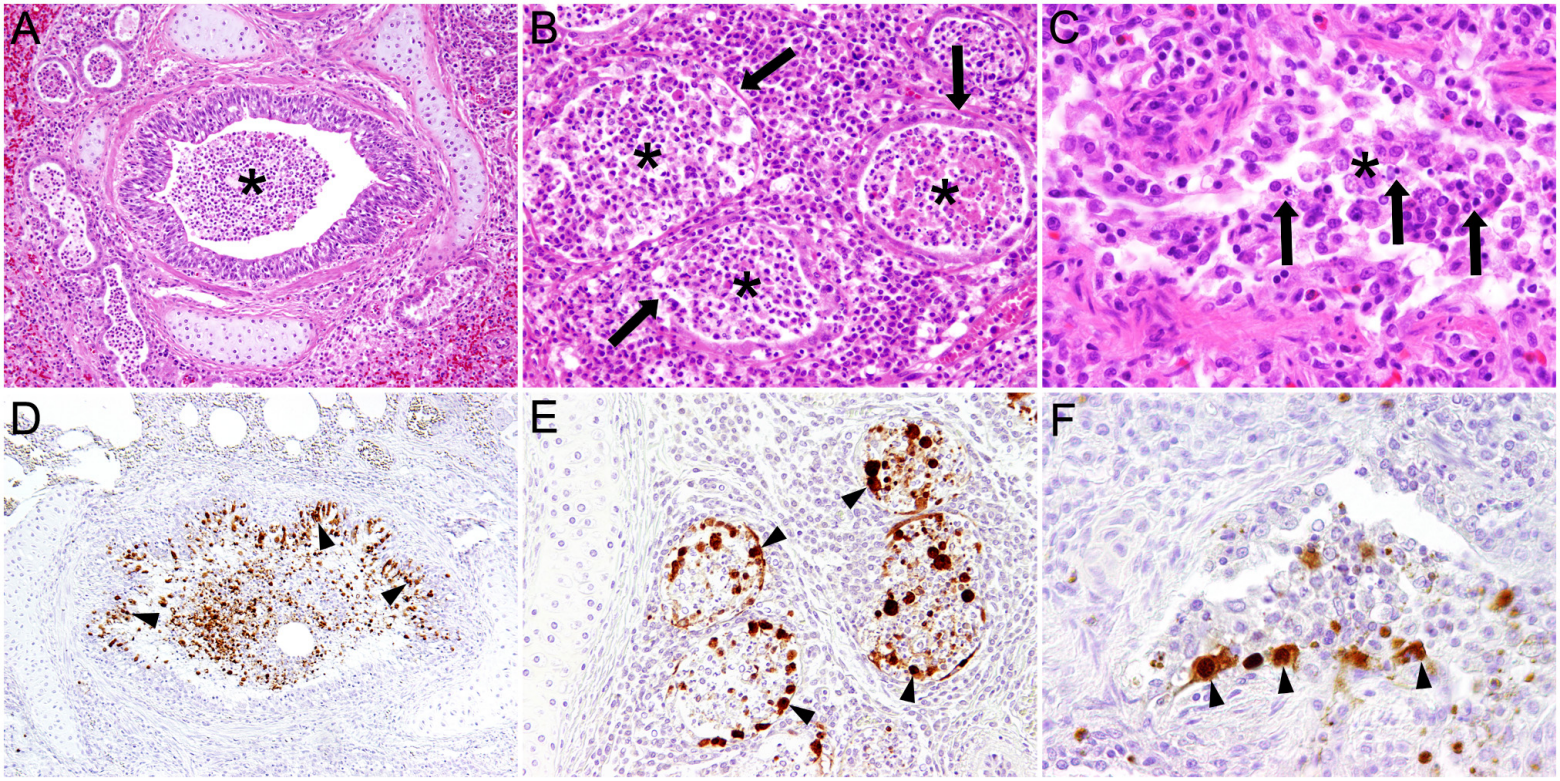
Histopathology and immunohistochemistry of bronchial, bronchiolar and bronchial glandular epithelium of a harbor seal (No. 13) naturally infected with Seal/H10N7. Histopathology showed necrotizing bronchitis with accumulation of cellular debris in the lumen (asterisk) (A; magnification 100x), bronchial adenitis with necrosis of glandular epithelial cells (arrows) and accumulation of cellular debris in the glandular lumen (asterisks) (B; magnification 200x) and bronchiolitis (C; magnification 400x) with accumulation of sloughed epithelial and inflammatory cells in the lumen (arrows). Immunohistochemistry revealed marked influenza A virus antigen in bronchial (arrowheads) (D; magnification 100x), bronchial glandular (arrowheads) (E; magnification 200x) and bronchiolar (F; magnification 400x) epithelial cells and intraluminal cells.

Histological examination revealed catarrhal rhinitis and tracheitis with epithelial single cell necrosis (Fig 1). In the lungs, acute necrotizing bronchitis, bronchiolitis and adenitis of bronchial glands with sloughing of epithelial cells were observed (Fig 2, Table 1). Occasionally, mild interstitial pneumonia was found. In addition, multifocal suppurative bronchopneumonia and pyogranulomatous to necrotizing pneumonia with intralesional adult nematodes and occasionally parenchymatous haemorrhages were observed. Furthermore, lungworms were also present in bronchi displaying epithelial and smooth muscle hyperplasia and within pulmonary lobules without inflammatory reaction.

Immunohistochemistry revealed specific staining of influenza A nucleoprotein exclusively in the respiratory tract (Figs 1 and 2, Table 1). The antigen was located in the nucleus and/or in the cytoplasm. Variable amounts of antigen were present in nasal and tracheal respiratory epithelial cells. Except for seal no. 13 with high numbers of infected cells in the nasal mucosa, the quantity of antigen-containing cells was low in the upper respiratory tract. Virus-specific staining in the lung was scattered. It was observed predominantly in respiratory epithelial cells of bronchi and epithelial cells of bronchial glands. In two animals (nos. 5 and 12) epithelial cells of single bronchioles were immuno-labelled and in seals nos. 4 and 9 single antigen-containing elongated cells resembling type I pneumocytes were found in the pulmonary parenchyma. Antigen was not detected in extra-respiratory tissues or organs including brain, lymphatic tissues, heart, liver, pancreas, digestive tract and uro-genital organs. Immunohistochemistry for morbillivirus antigen was negative in lungs and brains of all animals.

**Fig 3:**
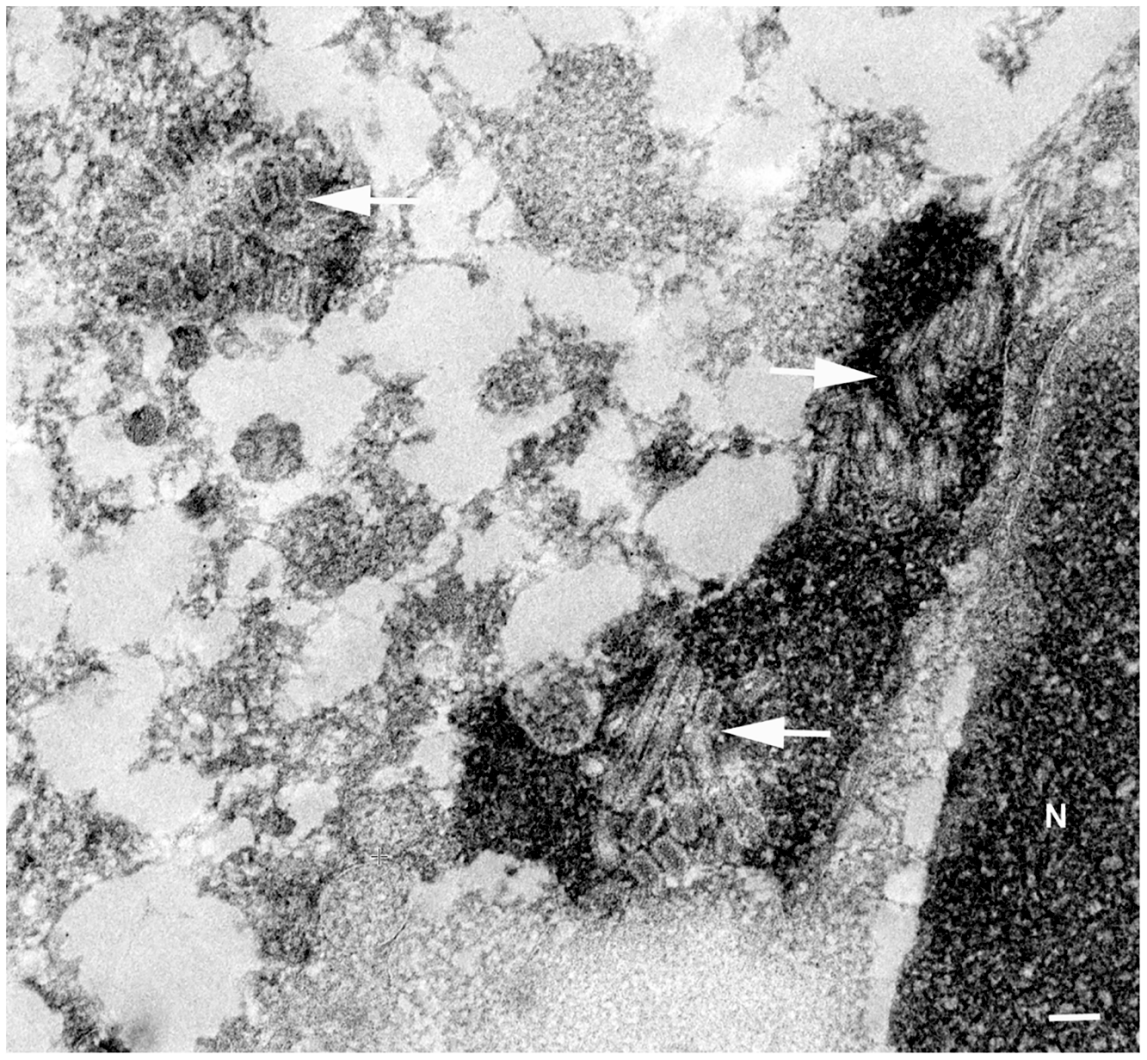
Ultrastructual image of virions in a bronchial epithelial cell of a harbor seal naturally infected with Seal/H10N7. Ultrastructural demonstration of aggregated spherical and tubular influenza virions (arrows) in the cytoplasm of a bronchial epithelial cell of seal no. 16; N = nucleus; pop-off technique applied on formalin-fixed, paraffin-embedded tissue section; N = nucleus; magnification 25.000x; bar = 100 nm.

Ultrastructurally, clusters of rod-shaped virus particles up to 300 nm long were found in the cytoplasm of bronchial epithelial cells (Fig 3).

Except for seal no. 10, in all lung samples a positive signal was detected by the influenza A matrix gene real-time RT-PCR. In nine examined tracheal swabs (seal nos. 8–16) and in three examined nasal swabs (seal nos. 13–15) a positive signal was detected by the influenza A matrix gene real-time RT-PCR. Lung tissue of all seals except no. 10 tested positive for influenza virus by RT-PCR. All swabs that were collected from seals—both tracheal swabs (seal nos. 8 to 16) and throat swabs (seal nos. 13 to 15)—tested positive for influenza virus by RT-PCR.

Microbiological investigation of the lung revealed in three seals (nos. 1, 8, 12) *Streptococcus equi* ssp. *zooepidemicus*, in three seals (nos. 3, 4, 7) *Bordetella bronchiseptica*, in seven seals (nos. 2, 6, 9, 10, 12, 13, 16) *Streptococcus phocae* (Table 1) and in one seal (no. 11) *Brucella* sp. In three seals (nos. 5, 14, and 15) a low number of various non-specific bacteria were isolated. In 4 (nos. 2, 3, 4, 7) out of 16 seals *Mycoplasma* spp. were identified. The findings of additional microbiological tests of other organs are compiled in (S1 Table).

Parasitologically, *Otostrongylus circumlitus* and *Parafilaroides gymnurus* were identified in the lungs of 14 seals. In seal no. 16, the heartworm *Acanthocheilonema* (previously termed *Dipetalonema*) *spirocauda* was additionally observed.

### Experimentally infected ferrets with Seal/H10N7

Six ferrets were inoculated intratracheally with H10N7 influenza virus A/harbor seal/Germany/PV20762_TS/2014 (Seal/H10N7). At each of days 3 and 7 post inoculation, three ferrets at each time point were euthanized. All animals survived and clinical examination of the ferrets demonstrated that they were less active starting at 3 days post inoculation (dpi) with ruffled fur, mild nasal discharge, half-closed eyes and activity score 1 (6 of 6 ferrets). At 4 dpi, the remaining three ferrets showed decreased activity with score 2 (1 of 3) or score 1 (2 of 3). At 5 dpi all three ferrets had score 1, at 6 dpi two ferrets had score 2 and one ferret was apathetic, shaking with increased abdominal breathing with score 3, and at 7 dpi the condition of the fur improved and all three ferrets had score 0. The ferrets lost between 4 and 6% of body weight at 3 dpi and between 8 and 18% of body weight at 7 dpi (S1 Fig).

Grossly, there was multifocal consolidation of the lung parenchyma that was red-grey at 3 dpi with 10–20% of the tissue affected and dark red at 7 dpi with 10–40% of the tissue affected. The median relative lung weight increased in time; at 3 dpi it was 0.90% (range 0.89–1.02) and at 7 dpi it was 1.32% (1.25–1.82). The tracheo-bronchial lymph nodes were enlarged in all animals; this enlargement was estimated to be 2 to 3 times normal size at 3 dpi and 4 times normal size at 7 dpi. Two ferrets had mild hepatic lipidosis (one ferret at 3 dpi and one at 7 dpi).

**Fig 4:**
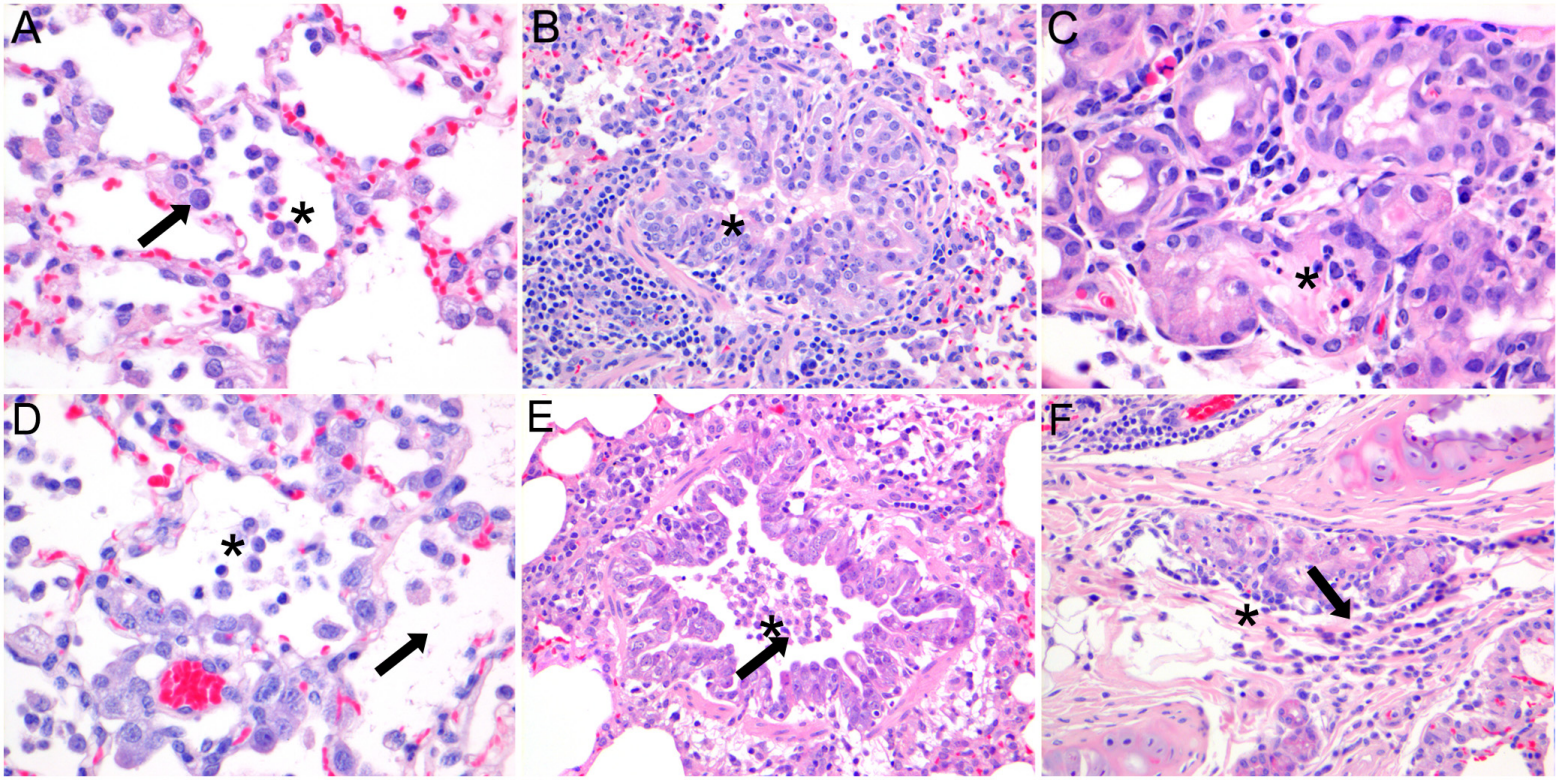
Histopathology of lungs of ferrets intratracheally inoculated with Seal/H10N7 at 3 and 7 dpi. Histopathology at 3 dpi showed multifocal mild to severe necro-suppurative alveolitis (A), bronchiolitis (B) and bronchial adenitis (C), characterized by mild epithelial necrosis, exocytosis of neutrophils, mild hypertrophy of epithelial cells (arrow), peribronchiolar infiltrates of lymphocytes, plasma cells and macrophages and accumulations of intraluminal neutrophils, macrophages and proteinaceous material (asterisk). At 7 dpi there was mild to severe necro-suppurative alveolitis (D), bronchiolitis (E) and bronchial adenitis (F). Compared to 3 dpi, at 7 dpi there was more severe hypertrophy of epithelial cells, there were less neutrophils and more intraluminal macrophages. (Magnification 400x (A, C, D), magnification 200x (B, E, F)).

Histopathology (Fig 4) at 3 dpi revealed multifocal mild neutrophilic infiltration in the epithelium (exocytosis) of the trachea and mild to severe suppurative and necrotizing bronchitis, bronchiolitis, bronchial adenitis, and bronchiole-centred alveolitis. The bronchitis, bronchiolitis and adenitis were characterized by mild epithelial necrosis, exocytosis of neutrophils, hypertrophy of epithelial cells and accumulations of intraluminal neutrophils, macrophages and proteinaceous material. In the peribronchial, peribronchiolar and perivascular interstitium there were multifocal mild to moderate infiltrates consisting of macrophages, lymphocytes, plasma cells and neutrophils, mild edema and mild to moderate proliferation of bronchus-associated lymphoid tissue. The alveolar septa were distended by infiltration of neutrophils, mild edema and had epithelial necrosis, in the alveolar lumina there were moderate numbers of neutrophils and alveolar macrophages mixed with proteinaceous material. In the tracheo-bronchial lymph nodes and spleen there was moderate lymphocytic hyperplasia and histiocytosis. At 7 dpi, the lesions in the lungs were similar but with fewer neutrophils and more macrophages and lymphocytes, hypertrophy and hyperplasia of bronchiolar epithelial cells, type II pneumocyte proliferation, intraluminal cellular debris and interstitial edema.

**Fig 5:**
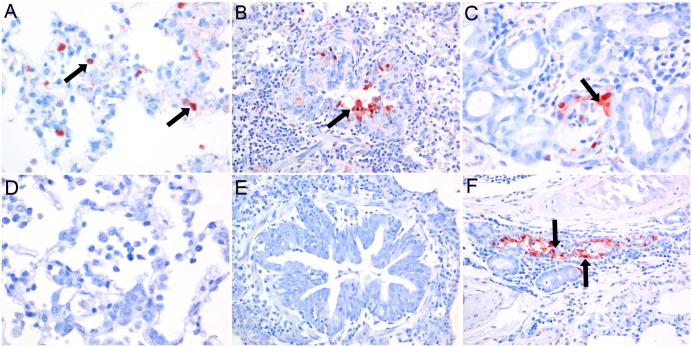
Immunohistochemistry of lungs of ferrets intratracheally inoculated with Seal/H10N7 at 3 and 7 dpi. Immunohistochemical staining at 3 dpi of influenza A virus antigen (arrows) was present in few type I and II pneumocytes (A), moderate numbers of bronchiolar epithelial cells (B) and few bronchial glandular epithelial cells (C). Compared to 3 dpi, immunohistochemical staining of influenza A virus antigen at 7 dpi was decreased or absent in alveolar (D) and bronchial epithelial cells (E), but similar in bronchial glandular epithelial cells (F). (Magnification 400x (A, C, D), magnification 200x (B, E, F)).

Immunohistochemical staining of influenza virus antigen (Fig 5) was associated with lesions and seen at 3 dpi in moderate numbers of bronchiolar epithelial cells (3/3), few bronchial glandular epithelial cells (2/3), few type I and II pneumocytes (3/3) and occasional bronchial and tracheal epithelial cells (2/3). At 7 dpi, staining of influenza virus antigen was present only in 2 ferrets in few bronchial and bronchial glandular epithelial cells (1/3) and occasional type II pneumocytes (1/3). No influenza virus antigen staining was present in the remaining respiratory or extra-respiratory tissues on both days.

**Fig 6:**
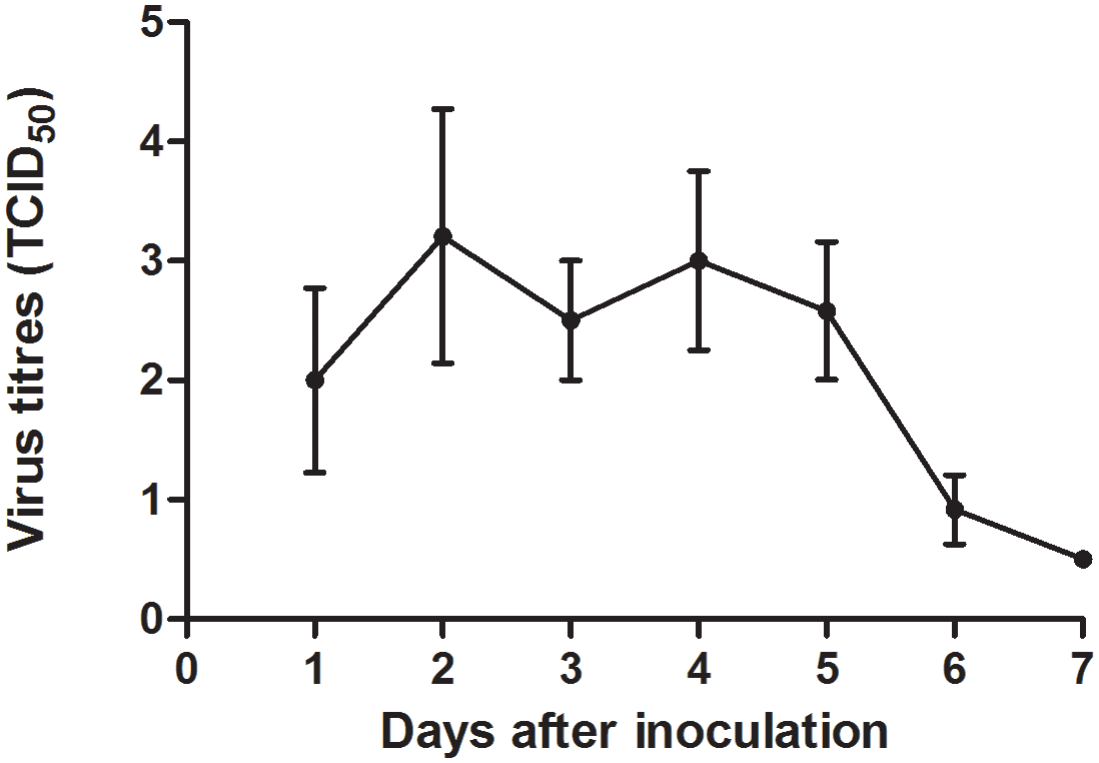
Average virus titers with standard deviation of pharyngeal swabs of ferrets intratracheally inoculated with Seal/H10N7. Virus was excreted in the pharynx from 1 to 6 dpi with highest values between day 2 and 4. Virus titers were measured in six ferrets from 1–3 dpi, and in three ferrets from 4–7 dpi. The lower limit of detection was 0.5 TCID_50_/ml.

Virologically, virus was detected in the pharyngeal swabs (Fig 6) from 1 to 7 dpi with a peak at 2 dpi (6/6). In nasal swabs, virus was isolated only in one ferret at 2 (2.25 TCID_50_/g) and 3 (1 TCID_50_/g) dpi. In the organs (Table 2) at 3 dpi, high virus titers were detected in lung (3/3), bronchus (3/3), trachea (3/3) and nose (1/3), low virus titers in tracheobronchial lymph node (1/3), tonsil (1/3) and olfactory bulb (2/3), and no virus in other extra-respiratory tissues. At 7 dpi, low virus titers were detected in lung (3/3), bronchus (2/3) and trachea (2/3), while no virus was isolated from the other respiratory or extra-respiratory tissues.

**Table 2:** Virus isolation from tissues in ferrets inoculated intratracheally with Seal/H10N7.

Tissues	3 dpi	7 dpi
**Nasal turbinates**	1/3 (8.6)*	0/3
**Trachea**	3/3 (4.0–4.9)	2/3 (3.6–4.1)
**Bronchus**	3/3 (3.3–4.7)	1/3 (4.3)
**Lung**	3/3 (5.3–6.6)	3/3 (1.6–4.0)
**Tracheobronchial lymph node**	1/3 (2.4)	0/3
**Tonsil**	1/3 (2.2)	0/3
**Olfactory bulb**	2/3 (2.1–3.9)	0/3

*Number of animals positive / total number or animals (virus titer; range log10TCID_50_/gram tissue). Cut off value: 0.5 TCID_50_/g.

## Discussion

In this study, we described histopathological changes, viral antigen distribution and cell type tropism after spontaneous infection of LPAI Seal/H10N7 in harbor seals and we compared lesions, viral antigen distribution and cell tropism in an animal model for human disease using ferrets after intratracheal inoculation with a Seal/H10N7 isolated from one of the harbor seals. Seal/H10N7 caused respiratory disease in both naturally infected wild harbor seals and experimentally infected ferrets. In both species, virus-induced lesions and cell type tropism were restricted to the respiratory tract with no evidence for spread to extra-respiratory tissues. In the respiratory tract of seals virus antigen and inflammation were most prominent in bronchial and submucosal glandular epithelial cells whereas in ferrets they were mainly found in bronchiolar and bronchial submucosal glandular epithelial cells. This slight difference in the predominant tissue structure tropism may depend on the species difference, perhaps a species-specific difference in cell-receptor distribution.

In the harbor seals, necrotizing rhinitis, tracheitis, bronchitis and bronchial adenitis were seen, associated with intralesional viral antigen labeling centered on bronchial and submucosal glandular epithelial cells and less in nasal and tracheal epithelial cells. In addition, necro-suppurative and/or granulomatous and eosinophilic inflammation was present most likely due to secondary bacterial infections and parasitic infestations. Our findings share morphological findings that have been described in seals infected with Seal/H10N7 from Sweden and Denmark, however, immunohistochemical staining for virus antigen has not been described [1, 2]. Lesions and cell type tropism of Seal/H10N7 infection in harbor seals is in accordance with previously described natural infections of harbor seals with LPAI H3N8 influenza virus in which virus and lesions were mainly present in the bronchi [7], and of harbor seals naturally infected with LPAI H7N7 that developed necrotizing bronchitis, bronchiolitis and hemorrhagic alveolitis [27]. The latter report mentioned concurrent infection with *Mycoplasma* sp. that has been shown in 25% of the investigated seals. However, *Mycoplasma* sp. was also detected in influenza negative seals (data not shown). Experimental infection with LPAI H7N7 demonstrated pneumonitis and virus isolation from the lung and bronchial lymph node without description of bronchitis [28].

The most severe lesions in the harbor seals infected with Seal/H10N7 were suppurative or pyogranulomatous bronchopneumonia caused by co-infections with bacteria and/or lungworms that most likely were the cause of death. Similar lesions caused by bacterial or parasitic infections represent common causes of death particularly in young harbor seals from the North Sea [29]. However, primary influenza A virus infection has led to an increased mortality in the seal population during the epidemic. Concurrent infections with bacteria were also described in Sweden and Denmark since all animals had severe growth in the lungs of *Pseudomonas aeruginosa*, *Streptococcus equi* ssp. *zooepidemicus* or *Escherichia coli* [1, 2]. The presence of severe lesions due to bacterial co-infections was also seen in the 1918 and 1957 influenza pandemics in humans since antibiotics were not available at that time [30]. Also in previous reports on influenza outbreaks in seals, concurrent infections with different bacteria (among which *Mycoplasma* spp., *Streptococcus* spp., *E*. *coli*, *Staphylococcus epidermidis* and *S*. *aureus*, *Clostridium* spp. and *C*. *perfringens*, *Neisseria* spp., *Klebsiella* spp., *Pseudomonas* spp., *Pasteurella* spp., *Aggregatibacter* [formerly known as *Actinobacillus*] *actinomycetemcomitans* and *Bordetella bronchiseptica*) were described, e.g. with H7N7 [27] or H3N8 [7]. It is noteworthy that *S*. *equi* ssp. *zooepidemicus* and *B*. *bronchiseptica* were isolated only from lungs of seals that died during phocine distemper or influenza epidemics [31–33], which may be explained by the immunosuppressive effects of morbilli- and influenza virus infections. During regular health monitoring of dead seals found stranded on the coast of Schleswig-Holstein, Germany, independent of mass mortalities, these bacteria were not found (unpublished data). Apparently, Seal/H10N7 by itself is not so virulent, but may be lethal in combination with bacterial infection as was seen in human fatal cases of 1918 influenza virus infection [34]. The lungworm infection associated with the (pyo)granulomatous pneumonia was most likely pre-existent because of the chronicity of the lesions. Furthermore, Geraci et al. showed that the prevalence of lung- and heart-worm infections in seals naturally infected with H7N7 influenza virus was not different from seals stranded due to other causes [27].

Virus infection, cell tropism and associated lesions of Seal/H10N7 infection were evaluated using intratracheally inoculated ferrets as an animal model for human disease (reviewed by van den Brand [35]). They were susceptible to infection and developed respiratory disease, which suggests that Seal/H10N7 is potentially virulent for human beings. Ferrets showed bronchopneumonia associated with intralesional virus antigen mainly located in bronchiolar epithelial cells and less frequently in type I and II pneumocytes, bronchial, bronchial glandular and tracheal epithelial cells. Also, virus was predominantly found in pharyngeal swabs, trachea, bronchus and lung but not in the extra-respiratory organs. In ferrets, the bronchiolar tropism of Seal/H10N7 was similar to that of influenza A[H1N1]pdm09 virus (H1N1 pandemic), and was associated with similar but less severe lesions; the tropism was different from HPAI H5N1, which is more present in the alveoli and accounts for more severe alveolitis than was seen in our ferrets [36]. This fits with the lower virulence seen in Seal/H10N7 infection when compared to H1N1 pandemic and HPAI H5N1 infections and suggests that Seal/H10N7 potentially may infect humans and cause a respiratory disease with similar, albeit less severe lesions compared to those seen in humans with H1N1 pandemic or HPAI H5N1 infections.

Interestingly, the severity of the lesions in the lungs of the ferrets was comparable or even more severe at 7 dpi than at 3 dpi despite lacking virus antigen expression. This may be caused by a delayed inflammatory response due to influx of inflammatory cells, response to cell necrosis and cytokine production after infection, even when there was no virus present in cells anymore (as reviewed by van den Brand [35]).

Sequence analyses indicated that Seal/H10N7 virus was most closely related to LPAIV [1–3, 5]. Humans infected with H10N7 from live poultry developed conjunctivitis and mild upper respiratory symptoms [37] and marine biologists working with stranded seals or with experimentally infected seals during the outbreak of LPAI H7N7 in 1979 developed purulent conjunctivitis associated with similar H7N7 influenza virus infection [38]. So far, no Seal/H10N7 infections have been recorded in people during this outbreak. However, this virus may again cross the species barrier and infect humans working with live (or dead) seals.

When comparing the histopathology of H10N7seal infection between the naturally infected harbor seals and the experimentally infected ferrets, it has to be considered that the time course of the natural infection is unknown. However, similar changes were seen in the lower respiratory tract including tracheitis, bronchitis, bronchial adenitis and to a lesser extent bronchiolitis. During the influenza outbreak there were also seals with a similar pattern of lesion that were negative for influenza A virus by PCR and immunohistochemistry (data not shown) possibly indicating virus clearance. When comparing cell type tropism, in both seals and ferrets tracheal, bronchial and bronchial glandular epithelial cells were positive for virus antigen. Virus attachment studies with LPAI viruses (H7N7 and H4N5 mallard) showed moderate virus attachment to trachea and bronchi, moderate to scarce attachment to bronchioles and scarce attachment to alveoli of harbor seals [39], which is consistent with the tropism found by IHC in the harbor seals naturally infected with Seal/H10N7. In contrast, human influenza viruses showed no attachment to trachea and bronchi and only scarce attachment to bronchioles and alveoli of harbor seals [39]. LPAI H5N9 and H6N1 in ferrets, like HPAI H5N1, showed attachment only to alveoli [40], which could explain the alveolitis but may not reflect the situation for H10N7, which also was present in trachea, bronchi and bronchioles of ferrets in our study.

The difference between the seals and the ferrets is the absence of lesions and virus antigen in the nose of the ferrets which may be in part related to the natural infection in harbor seals versus the intratracheal inoculation of the ferrets. Since the ferrets were inoculated intratracheally to evaluate the pulmonary lesions and virus titers, presence of virus in the nose was very inconsistent and therefore not conclusive for virus replication and lesions in the nose.

Another difference is that the seals had virus antigen presence in occasional type I pneumocytes and the ferrets in few type I and II pneumocytes which may reflect a slight difference in tropism between the two species. The rarity of virus antigen presence in alveolar epithelial cells in the seals may reflect the low tropism of Seal/H10N7 for alveolar epithelial cells, it may be related to the time-related clearance after infection as was seen in ferrets with less cells positive for antigen at 7 dpi, or it may be related to masking due to bacterial co-infection, which was not seen in the experimentally infected ferrets. In one ferret with influenza virus in the olfactory bulb, contamination during necropsy from the adjacent virus-positive nasal cavity cannot be excluded.

In both the naturally infected harbor seals and the experimentally infected ferrets, virus antigen was seen in bronchial glands with associated bronchial adenitis. Similar tropism for bronchial glands with subsequent lesions has been described for H1N1pandemic infection in ferrets and for seasonal human influenza virus H1N1 and H3N2 infections in humans [35]. Also, virus attachment studies demonstrated tropism of seasonal human influenza virus and avian LPAI and HPAI viruses for bronchial submucosal glands in humans [41] and of H1N1 pandemic and seasonal human influenza virus in ferrets [40]. Tropism for bronchial glandular epithelial cells may play a role in efficient mammal-to-mammal transmission, since virus excreted from these glands into the bronchus is more likely to be expectorated than virus produced in the alveolar epithelial cells.

In conclusion, Seal/H10N7 infection caused mild to moderate inflammation in the upper and lower respiratory tracts of naturally infected harbor seals together with concurrent bacterial infection that resulted in fatal pneumonia. Additionally, intratracheally infected ferrets had moderate inflammation in the lower respiratory tract, indicating that humans may potentially be infected and develop disease when infected with Seal/H10N7. This mass outbreak of H10N7 in seals demonstrates the ease of a bird-to-seal infection of a low pathogenic avian influenza virus such as this Seal/H10N7 from wild birds and thus be a potential risk for humans. In case of a future outbreak in harbor seals caused by Seal/H10N7 or a related virus, its zoonotic potential needs to be evaluated and taken into account.

## Supporting Information

S1 Table Microbiological findings in liver, spleen, kidney, intestine, pulmonary and mesenteric lymph nodes of seals naturally infected with Seal/H10N7. (DOCX)

S1 Fig Body weight loss (%) of ferrets intratracheally inoculated with Seal/H10N7. The body weight loss from 1 to 3 dpi was more severe than that from 4 to 7 dpi.
(TIF)
