# Supplementary material for: Influenza A (H10N7) Virus Causes Respiratory Tract Disease in Harbor Seals and Ferrets
Source: PLoS One. 2016 Jul 22;11(7):e0159625. doi: 10.1371/journal.pone.0159625 (PMC4957826; doi:10.1371/journal.pone.0159625)
Supplement: S1 Table — (DOCX) [file pone.0159625.s001.docx]

**Table S1. Microbiological findings in liver, spleen, kidney, intestine, pulmonary and mesenteric lymph nodes of seals naturally infected with Seal/H10N7.**

| **Animal**  **No.** | **VirologyNo.** | **Liver** | **Spleen** | **Kidney** | **Lymph node**  **(pulmonary)** | **Intestine** | **Lymph node (intestinal)** |
| --- | --- | --- | --- | --- | --- | --- | --- |
| **1** | PV20731 | +++ S. equi ssp. zooepidemicus; | +++ S. equi ssp. zooepidemicus; | +++ S. equi ssp. zooepidemicus; | +++ S. equi ssp. zooepidemicus; | +++ S. equi ssp. zooepidemicus;  +++ Cl. perfringens;  + Proteus sp.;  + Moellerella wisconsenis; | +++ S. equi ssp. zooepidemicus; |
| **2** | PV20737 | ++ S. phocae;  + Staphylococcus sp.; | + S. phocae;  + Staphylococcus sp.; | ++ S. phocae; | +++ S. phocae;  + Staphylococcus sp. | + Proteus sp.; | ++ S. phocae;  + Lactobacillus sp.;  + α-hem. S.; |
| **3** | PV20738 | + E. coli; | Almost no bacterial growth; | Almost no bacterial growth; | + E. coli; | ++ E. coli;  ++ hem. E. coli; | + E. coli;  ++ α-hem. S. |
| **4** | PV20739 | + E. coli; | ++ E. coli; | ++ E. coli; | + E. coli;  + B. bronchiseptica;  + Moraxella sp. | + E. coli; | + E. coli; |
| **5** | PV20742 | + E. coli; | + E. coli; | ++ E. coli; | + E. coli; | +++ E. coli;  +++ Cl. perfringens; | + E. coli;  + coli-like germs; |
| **6** | PV20743 | + E. coli; | Almost no bacterial growth; | + E. coli; | + B. bronchiseptica; | + S. phocae;  +++ E. coli;  + B. bronchiseptica;  + Vibrio sp.; | +++ E. coli; |
| **7** | PV20741 | + E. coli;  + Psychrobacter sp.; | Almost no bacterial growth; | ++ Moraxella sp.;  ++ E. coli;  + Proteus sp.;  + Psychrobacter sp.; | + E. coli;  + B. bronchiseptica; | + Moraxella sp.;  ++ E. coli;  + Psychrobacter sp.; | + Moraxella sp.;  ++ E. coli; |
| **8** | PV20763 | +++ S. equi ssp. zooepidemicus; | +++ S. equi ssp. zooepidemicus; | +++ S. equi ssp. zooepidemicus;  + Kluyvera sp.; | +++ S. equi ssp. zooepidemicus;  + Kluyvera sp.;  ++ Macrococcus sp.; | +++ S. equi ssp. zooepidemicus; | +++ S. equi ssp. zooepidemicus; |
| **9** | PV20762 | Almost no bacterial growth; | + coli-like germs; | Almost no bacterial growth; | + Brucella sp.;  + Moraxella sp.; | + S. equi ssp. zooepidemicus;  + Pantoea sp.; | + Brucella sp.; |
| **10** | PV20745 | Almost no bacterial growth; | Almost no bacterial growth; | Almost no bacterial growth; | + Stenotropho-monas maltophilia;  + S. phocae; | ++ Enterococcus sp.;  +++ S. phocae;  + Acinetobacter sp.;  + Serratia liquefaciens;  +++ Cl. perfringens; | No bacterial growth; |
| **11** | PV20764 | No bacterial growth; | No bacterial growth; | No bacterial growth; | + Neisseria sp.; | + S. equi ssp. zooepidemicus; | Almost no bacterial growth; |
| **12** | PV20744 | No bacterial growth; | Almost no bacterial growth; | + S. equi ssp. zooepidemicus;  + S. phocae; | + S. equi ssp. zooepidemicus;  + S. phocae;  + Moellerella wisconsenis;  + Neisseria sp.;  + Arcanobacterium phocae; | ++ S. equi ssp. zooepidemicus;  + E. coli; | + S. equi ssp. zooepidemicus; |
| **13** | PV20769 | Almost no bacterial growth; | + α-hem. S.; | Almost no bacterial growth; | Almost no bacterial growth; | No bacterial growth; | No bacterial growth; |
| **14** | PV20768 | ++ Arcano-bacterium phocae;  + Neisseria sp.;  + α-hem. S.;  + γ-hem. S.; | + Arcanobacterium phocae;  + Neisseria sp.;  + γ-hem. S.;  + S. phocae; | +++ Arcano-bacterium phocae;  ++ Neisseria sp.;  + Corynebacterium sp.; | + Arcanobacterium phocae;  + Neisseria sp.;  + α-hem. S.;  + γ-hem. S.;  + Corynebacterium sp.; | + Arcanobacterium phocae;  + Neisseria sp.; | + Arcanobacterium phocae;  + Neisseria sp.; |
| **15** | PV20767 | Almost no bacterial growth; | Almost no bacterial growth; | + γ-hem. S.;  + Arthrobacter sp.; | Almost no bacterial growth; | + γ-hem. S.;  +++ Cl. perfringens; | Almost no bacterial growth; |
| **16** | PV20766 | ++ E. coli;  + Acinetobacter pittii; | ++ E. coli;  ++ Acinetobacter pittii; | ++ E. coli;  ++ Pseudomonas sp.;  + Myroides odoratus; | ++ E. coli;  + Arcanobacterium phocae;  + Stenotropho-monas maltophilia; | ++ E. coli;  ++ Acinetobacter pittii;  ++ Cl. perfringens;  + yeasts; | ++ E. coli;  + Acinetobacter pittii; |

S. = Streptococcus; E. = Escherichia; B. = Bordetella; Cl. = Clostridium; hem. = hemolysing

Almost no bacterial growth = < 5 colonies

+ = low numbers of bacteria (5-50 colonies)

++ moderate numbers of bacteria (51-250 colonies)

+++ high numbers of bacteria (> 250 colonies)
