# Supplementary figures and images for: Influenza A (H10N7) Virus Causes Respiratory Tract Disease in Harbor Seals and Ferrets
Source: PLoS One. 2016 Jul 22;11(7):e0159625. doi: 10.1371/journal.pone.0159625 (PMC4957826; doi:10.1371/journal.pone.0159625)

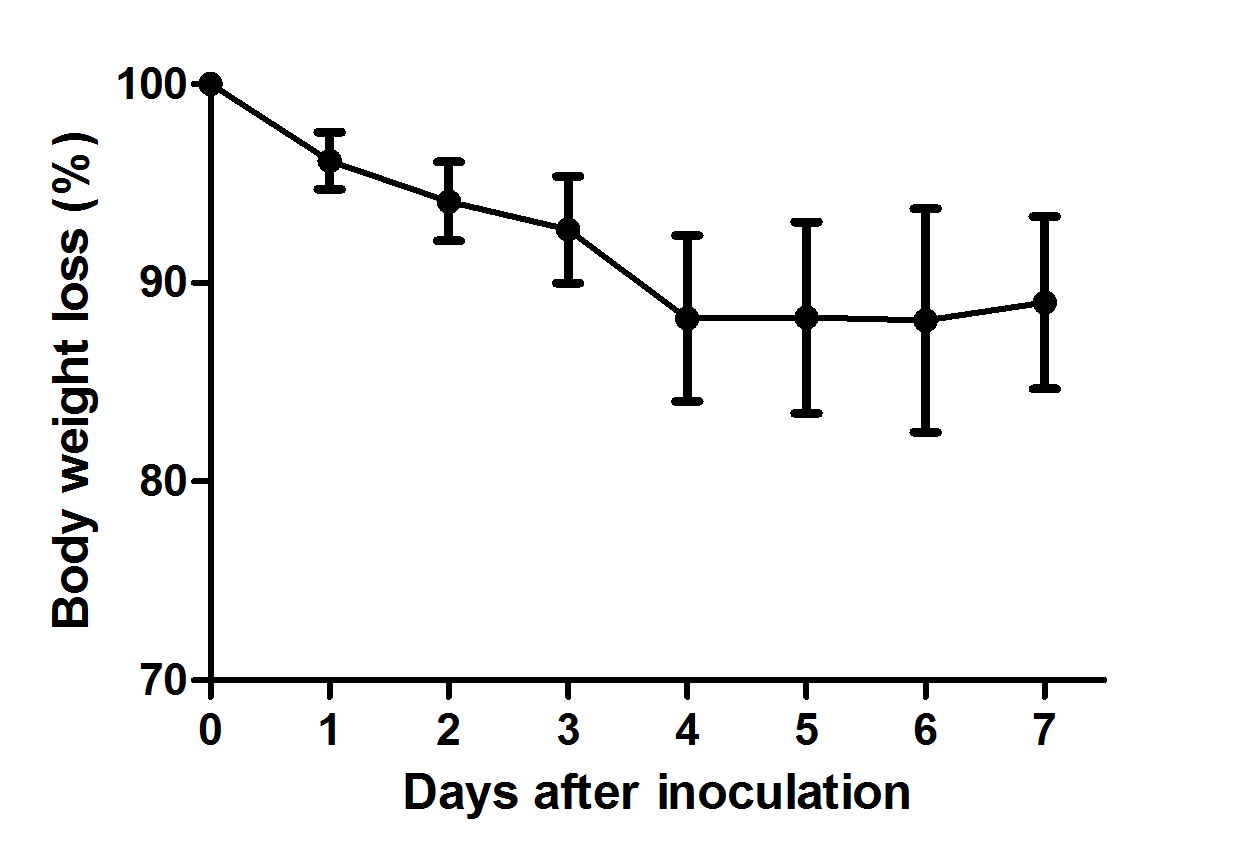

Supplement: S1 Fig — The body weight loss from 1 to 3 dpi was more severe than that from 4 to 7 dpi. (TIF) [file pone.0159625.s002.tif]
